# *In vitro* and *in vivo* evaluation of the genotoxicity of titanium dioxide, GST

**DOI:** 10.5620/eaht.2023008

**Published:** 2023-05-03

**Authors:** Ji-Soo Kim, Myung-Hwan Jeong, Heung-Sik Seo, Myeong-Kyu Park, Hee Ju Park, Seong-Soon Nah

**Affiliations:** 1 Healthcare advanced Chemical Research Institute, Korea Testing and Research Institute (KTR), Hwasun-gun, Jeollanam-do, Republic of Korea; 2 Research Laboratory, Bentech Frontier Co. Ltd., Nam-myeon, Jangseong, Jeollanam-do, Republic of Korea; 3 Division of Environment & Health, Korea Testing & Research Institute, Gwacheon-si, Gyeonggi-do, Republic of Korea

**Keywords:** TiO_2_, genotoxicity, bacterial reverse mutation test, chromosome aberration test, micronucleus test

## Abstract

Titanium dioxide (TiO_2_) was used in various applications in a wide range of products including food, cosmetics and photocatalyst. General toxicity studies of titanium dioxide, GST (Green Sludge Titanium) have been investigated in several reports, whereas studies concerning mutagenicity and genotoxicity have not been elucidated. Herein, we investigated the potential mutagenicity and genotoxicity of GST by genetic toxicology testing. The bacterial reverse mutation test was conducted by the pre-incubation method in the presence and absence of metabolic activation system (S9 mixture). The chromosome aberration test was performed using cultured Chinese hamster lung cell line in the absence and presence of S9 mixture. The micronucleus test was performed by using specific pathogen-free male ICR mice. Genotoxicity tests were conducted following the test guidelines of the Organisation for Economic Cooperation and Development with application of Good Laboratory Practice. No statistically significant increases were found in the bacterial reverse mutation test, in vitro chromosome aberration test, and in vivo micronucleus test when tested for induction of genotoxicity in GST. These results suggest that GST did not induce mutagenicity and genotoxicity in both in vitro and in vivo system.

## Introduction

Titanium dioxide (TiO_2_) are among the most widely produced and used photocatalyst in the world [1]. Titanium dioxide (TiO_2_) is an oxide of titanium that exists in nature in different crystalline forms; the most common form is rutile, whereas anatase and brookite are comparatively rare forms. These materials possess some unique properties for which they are used in many products; however, these same properties could also be the source of potential harm to biological systems, causing adverse effects [2]. In places like laboratories or industries where these TiO_2_ are produced, handled or processed, the workplace exposure can occur. Workers involved in TiO_2_ production can be exposed to TiO_2_ dust, the human body is exposed to TiO_2_ through four possible routes: inhalation, ingestion, skin penetration, and injection of nanomaterials as in nanomedicine [3]. A review of TiO_2_ genotoxicity data summarizing both in vivo and in vitro results was last published in 2014 [4]. In the present article, we have gathered and discussed the recent genotoxicity data of TiO_2_ and in vitro and in vivo genotoxicity studies are summarized.

The aim of this study was to determine the potential mutagenicity and genotoxicity of titanium dioxide, GST (Green Sludge Titanium). For accurate evaluation of genotoxicity, three types of in vitro and in vivo genotoxicity tests, i.e., the bacterial reverse mutation test, in vitro chromosomal aberration test, and in vivo micronucleus test were performed in compliance with the Organisation for Economic Cooperation and Development (OECD) guidelines for the testing of chemicals under the modern Good Laboratory Practice Regulations [5]. The results of this investigation will provide additional information relevant to the safety evaluation of titanium dioxide, GST exposure.

## Materials and Methods

### Bacterial reverse mutation test

The bacterial reverse mutation test was conducted in accordance with the OECD guideline No. 471 for the testing of chemicals [6]. The tester strains purchased from Molecular Toxicology Inc. (Boone, NC, USA) used in this study were Salmonella typhimurium TA98, TA100, TA1535, and TA1537, and Escherichia coli WP2uvrA in the absence and presence of metabolic activation system. The metabolic activation system was prepared by mixing S9 metabolic activation (Molecular Toxicology Inc.) with Cofactor 1 from Wako Pure Chemical Industries Ltd (Osaka, Japan), giving a final concentration of 5% (volume/volume) S9. The tester strains were cultured in 2.5% nutrient broth No. 2 (Oxoid Ltd, Basingstoke, UK) in a 37 °C shaking incubator (120 rpm) for approximately 10 h. The mutagenicity test was performed by mixing test substance and tester strains, which was cultured overnight in the presence and absence of the S9 mixture. Next, the mixture was incubated in a water bath for 20 min at 37 °C, mixed with top agar and a minimal amount of histidine-biotin (for S. typhimurium strains) or tryptophan (for E. coli strain), and then poured onto the surface of a gamma-ray sterile Falcon® Petri dish (Thermo Fisher Scientific, Waltham, MA, USA) containing about 15 mL of minimal glucose agar. The finished plates were incubated for 72 h at 37 °C. The number of revertant colonies was then counted. Three plates per treatment group were conducted, and the results were tabulated as the mean ± standard deviation for each condition.

### *In vitro* chromosomal aberration test

The clastogenicity of GST was evaluated for its ability to induce chromosomal aberrations in Chinese hamster lung (CHL) fibroblast cells. The chromosomal aberration test was conducted in accordance with the OECD guideline No. 473 for the testing of chemicals [7]. A clonal subline of CHL cells was obtained from the American Type Culture Collection (Rockville, MD, USA). The karyotype of the CHL cells consisted of 25 chromosomes. The CHL cells were grown in Minimum Essential Eagle’s Medium, supplemented with 10% fetal bovine serum (FBS), 50 U/mL penicillin, and 50 μg/mL streptomycin (Gibco BRL Life Technologies Inc., Gaithersburg, MD, USA) at 37 °C in a humidified atmosphere containing 5% CO_2_. Short-time treatment method (with or without metabolic activation system) and continuous treatment method were conducted. Mitomycin C (Sigma-Aldrich Co., St Louis, MO, USA) was used as a positive control, both with and without the S9 mixture. After 22 h of incubation, colcemid was added to the cultures at a final concentration of 0.2 μg/mL, and meta phase cells were harvested by trypsinization and centrifugation. The cells were swelled by adding hypotonic (0.075 M) KCl solution for 20 min at 37 °C, and then washed two times in ice-cold fixative (ethanol to glacial acetic acid, 3:1). A few drops of cell pellet suspension were dropped onto precleaned glass microscope slides and air-dried. The slides were stained with 5% Giemsa buffer solution (Thermo Fisher Scientific, Waltham, MA, USA). The number of cells with chromosomal aberrations was recorded on 300 well spread metaphases. Aberration frequencies, defined as aberrations observed, were divided by the number of cells counted, and were analyzed using Fisher’s exact test with Dunnett’s adjustment.

### *In vivo* micronucleus test

The micronucleus test was performed in accordance with the OECD test guideline No. 474 for the testing of chemicals [8]. The 7-week-old ICR mice were randomized into groups containing five mice each. As a result of the vehicle test, the test substance was suspended in sterile distilled water at 200 mg/mL, and there was no heat, foaming, or discoloration when mixed, so sterile distilled water was selected as the vehicle. The test substance was administered orally in three doses in volumes of 10 mL/kg. It was given twice with a 24 h interval in between, and test subjects were sacrificed by CO_2_ inhalation. The bone marrow was flushed with FBS (Gibco BRL Life Technologies Inc.). The centrifuge tubes were selected for centrifugation of the cells and centrifuged at 1000 rpm for 5 min. The suspended cells were smeared on a clean slide. The smeared slides were air-dried and thereafter fixed for 5 min in methanol. The 40 μg/mL acridine orange was dropped on the methanol-fixed slides for the observation.

In scoring the preparations, micronuclei were counted in polychromatic erythrocytes (PCE) and separately in monochromatic erythrocytes. The rate of micronucleated cells, expressed as a percentage, was based on the total of PCE present in the scored optic fields. The scoring of micronucleated normocytes was used to recognize the presence of artifacts (which is rare in mouse preparations), which provided additional interesting information on the mode of action of the test substances. Generally, an incidence of more than one micronucleated normocyte per 1000 PCE indicates an effect on cell stages, especially post-S-phase.

### Test procedure

#### Properties of GST

**Figure 1.**
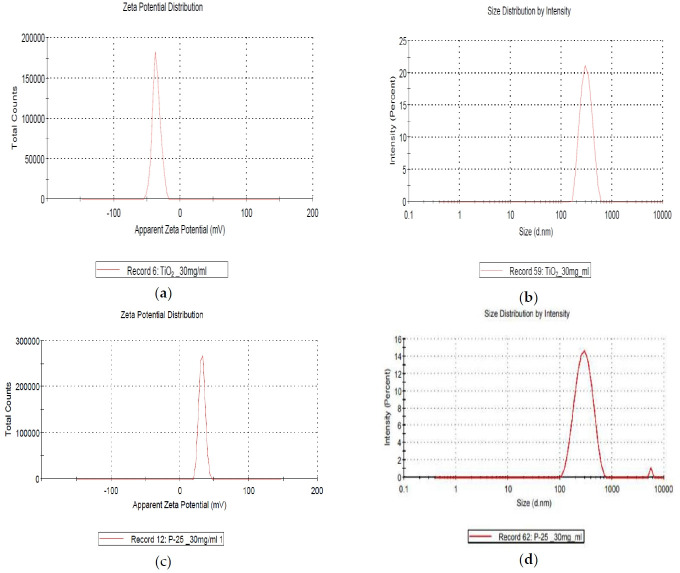
Characterization of TiO_2_ particles (GST) compared to P-25 analyzed by Korea TECH: (a) negative zeta potential (-35.4 mV, 30 mg/mL); (b) size distribution by intensity (mean: 336.8 nm); (c) positive zeta potential (32.3 mV, 30 mg/mL); (d) size distribution by intensity (mean: 302.2 nm).

**Figure 2.**
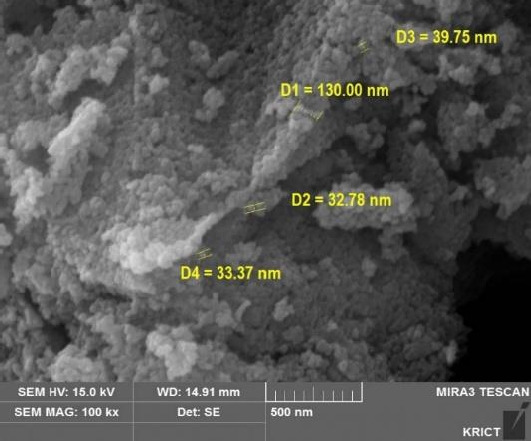
Characterization of TiO_2_ particles (GST): SEM (scanning electron microscope) image analyzed by KRICT.

**Figure 3.**
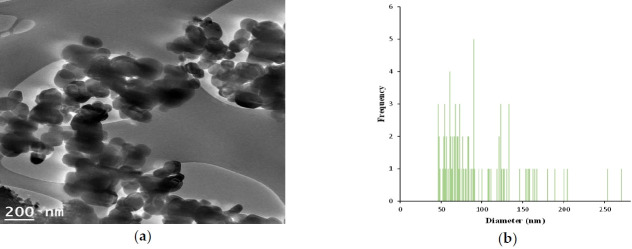
Characterization of TiO_2_ particles (GST); (a) A particles dispersed in 99.9 % EtOH was deposited on a copper grid and analyzed using TEM (Transmission electron microscope) image by Korea Basic Science Institute, (b) Size distribution (95.8 ± 46.3 nm, 46 – 270 nm,) of the imaged GST(image J software).

The characterization of GST is shown in Figure 1, 2 and 3, and individually compared to another titianium dioxide, P-25. The zeta potential is the potential between droplet surface and dispersing liquid medium and can be used to estimate surface charge of the droplets in the dispersion medium. Also, it was well known as indicator of the droplet stability, where values more positive than +30 mV and more negative than -30 mV indicate good stability against coalescence [9]. The estimated value for GST showed that GST has a negative value (-35.4 mV) thought to be good stability and it was thought to be a property to be less agglomeration nature (Figure 1). The particle size and distribution (95.8 ± 46.3 nm, 46 – 270 nm) showed that GST was considered to have materials of various sizes and was difficult to be classified as a nanomaterial considering the definition of nanomaterials (< 100 nm) (Figure 2–3).

#### Animal husbandary and maintenance

The 6-week-old male ICR mice were obtained from the Orient Bio Inc. (Gyeonggi, Korea). The animals were used after 7 days of acclimatization and housed animal to a stainless wire cage in a room with barrier system controlled for the light-dark cycle (12–12 h), air exchange (10–20 changes/h), temperature (19–25 °C) and relative humidity (30–70%) during the study. The animals were fed with Rodent Diet 20 5053 (LabDiet, USA) and provided with the reverse osmosis water ad libitum. The animals were maintained in accordance with the Guide for the Care and Use of Laboratory Animals [10].

#### Clinical signs and Body weight

During the study, all animals were observed once daily after treatment for any clinical signs of toxicity and mortality. The body weight of each mouse was measured at the time of animal receipt, grouping, before administration and autopsy.

#### Statistical analysis

The result of the statistical evaluation was deemed to be statistically significant when the P-value was less than 0.05. We used the Kruskal–Wallis H test and Dunnett’s test for differences in numbers of micronucleated polychromatic erythrocytes (MNPCE) between the treated and negative control groups; the Mann–Whitney U test for differences in numbers of MNPCE between the positive and negative control groups; analysis of variance and Dunnett’s test for differences in the PCE/(PCE + normochromatic erythrocyte (NCE)) ratio between the treated and negative control groups; the Student’s t-test for differences in the PCE/(PCE + NCE) ratio between the positive and negative control groups; and analysis of variance and Dunnett’s test for comparison of animal body weight at the time of euthanasia.

## Results and Discussion

### Bacterial reverse mutation test

In the concentration range-finding test (data not shown), it was found to have a nontoxic effect in all tester strains of S. typhimurium (TA98, TA100, TA1535, and TA1537) and in E. coli WP2uvrA at a dose of 5000 μg per plate in the presence and absence of S9 mixture. Based on the data from the concentration range-finding test, we selected 5000 μg per plate as the highest dose. As shown in Table 1, none of the tester strains showed any increase in the number of revertant colonies in comparison with the negative control group when the bacteria were treated with GST at 312.5, 625, 1250, 2500, and 5000 μg per plate, regardless of metabolic activation. On the other hand, the positive control group used in the assays with the presence or absence of S9 mixture showed positive responses by the respective test strains, as evidenced by the number of revertant colonies being greater than 2-fold of the respective those of negative control group. The bacterial reverse mutation test detects point mutations, which caused many human genetic diseases and play an important role in tumor initiation and development [11]. This assay is a widely accepted short-term test to identify substances that can produce genetic damage leading to gene mutations [12]. In the present study, we found no positive mutagenic responses to GST in any of the tester strains compared with the negative control both with and without the S9 mixture.

### *In vitro* chromosomal aberration test

Initially, a concentration range-finding test was performed to determine the test doses for use in the in vitro chromosome aberration test. The highest concentration was determined to be 50 μg/mL in a short-term treatment assay in the absence of S9 mixture (referred to as –S9 mix) and to be 80 μg/mL in the presence of S9 mixture (referred to as +S9 mix), to be 30 μg/mL continuous treatment test (referred to as 24 h exposure). The in vitro chromosome aberration test was conducted with GST at concentrations of 0, 12.5, 25 and 50 μg/mL without metabolic activation. In the presence of S9 mixture, the concentrations were 0, 20, 40 and 80 μg/mL. Except for the positive control group, cells arrested in metaphase with structural aberrations were less than 5% (Table 2). Chromosome aberrations are the classical genotoxic response to tumor initiation and development processes [13]. The purpose of the in vitro chromosome aberration test is to identify agents that cause structural chromosome aberrations in cultured mammalian cells [14]. Our results indicated no significant increase in the number of metaphases with structural aberrations at the four concentrations in GST, regardless of metabolic activation. The results of the in vitro chromosomal aberration test suggest that GST was not considered to have the ability to induce the chromosomal aberrations in CHL/IU cells under the present experimental conditions.

### *In vivo* micronucleus test

Based on the results of a dose range-finding test (data not shown), the highest dose of test substance in main test was determined at 2000 mg/kg body weight. The PCE/(PCE + NCE) ratios were used as an index of bone marrow cytotoxicity. The ratios did not show any significant difference in the GST treatment groups in comparison with the negative control group. The MNPCE frequencies were not statistically significant and did not show any dose-dependent pattern among the three treatment groups in comparison with the negative control group. On the other hand, the positive control group showed a significantly increased MNPCE frequency in comparison with the negative control group and GST treatment groups (Table 3). In all groups of main test, there was no dead animal during the study period and no significant difference in body weights compared with the negative control group (Table 4). The micronucleus test (MNT) is a useful assay for the detection of mutagenic substances, thus altering the equitable distribution of chromosomes during cell division [15]. The present micronucleus test was performed using the bone marrow cells of specific pathogen-free male ICR mice. GST did not induce any significant increases in MNPCEs, and there was no significant decrease in the PCE/(PCE+NCE) ratio up to 2000 mg/kg body weight in the GST treatment groups compared with the negative control group. From these results, GST was determined not to induce an increased frequency of micronuclei in the bone marrow cells of male ICR mice under the present experimental condition. Also, GST had no cytotoxicity effects in the bone marrow cells.

## Conclusions

Titanium dioxide (TiO_2_) is a natural oxide of the elements titanium with low toxicity, and negligible biological effects. In spite of the extensively use of TiO_2_, the biological effects are still not completely elucidated [16]. To determine the safety of TiO_2_ in the human health, systemic toxicological studies must be performed using various experimental models to predict the potential toxicity. Genotoxicity tests have been used mainly for the prediction of in vivo genotoxicity and carcinogenicity of chemicals, because compounds that show positive results in these tests have carcinogenic and/or mutagenic potential in humans [17]. In this study, the potential genotoxicity of the GST was examined in the three battery of in vitro and in vivo genotoxicity tests (bacterial reverse mutation test, chromosome aberration teat, and bone marrow micronucleus test) in accordance with the OECD Test Guidelines and Principles of Good Laboratory Practice.

The bacterial reverse mutation test detects point mutations, which caused many human genetic diseases and play an important role in tumor initiation and development [11, 18]. This test is a widely accepted short-term test to identify substances that can produce genetic damage leading to gene mutations [12, 19]. In the present study, we found no positive mutagenic responses to GST in any of the tester strains compared with the negative control, both with and without the S9 mixture.

Chromosome aberrations are the classical genotoxic responses to tumor initiation and development processes [13]. The purpose of the in vitro chromosome aberration test is to identify agents that cause numerical and structural chromosome aberrations in cultured mammalian cells [14]. Our results showed no significant increase in the number of metaphases with numerical and structural chromosome aberrations at the three concentrations of GST tested, regardless of metabolic activation. Based on the results of the in vitro chromosome aberration test, GST was not considered to have the ability to induce chromosomal aberrations in CHL cells under the present experimental conditions.

The micronucleus test detects mutagenic substances that alter the equitable distribution of chromosomes during cell division [15, 20]. The present micronucleus test was performed using the bone marrow cells of specific pathogen-free male ICR mice. GST did not induce any significant increases in MNPCEs, and there was no significant decrease in the PCE/(PCE+NCE) ratio up to 2000 mg/kg body weight in the GST treatment groups compared to that of the negative control group. From these results, GST was determined not to induce an increased frequency of micronuclei in the bone marrow cells of male ICR mice under the present experimental conditions. In addition, GST showed no cytotoxic effects in the mouse bone marrow cells.

TiO_2_ material, which is covered in this paper, GST (100% anatase) was prepared from the precipitated sludge using TiCl4 used as a coagulant to remove total phosphorus in the wastewater. The experimental genotoxicity data were restricted to studies from the largely applied comet assays, which is not validated by the OECD yet. In vivo data are also available for TiO_2_ materials, even if in vivo data are considered of higher relevance than in vitro data to conclude on genotoxicity, they are quite limited.

In conclusion, GST was regarded to have no genotoxic or cytotoxic potential in both in vitro and in vivo system under our current experimental condition. These results suggest that GST is relatively safe on the mutagenicity and genotoxicity, and are expected to provide some information on the risk assessment process.

## Figures and Tables

**Table 1.** Numbers of revertant colonies induced by GST in *Salmonella typhimurium* (TA98, TA100, TA1535, TA1537) and *Escherichia coli* (*WP2uvrA*) with and without metabolic activation (S9 mixture).

Tester Strain	Chemical treated	Dose (μg/plate)	Colonies/plate [Factor]^1^											
			Without S9 mixture						With S9 mixture					
TA98	GST	0	26	±	3				32	±	4			
		312.5	27	±	3	[	1.0	]	35	±	2	[	1.1	]
		625	28	±	1	[	1.1	]	35	±	4	[	1.1	]
		1250	25	±	5	[	0.9	]	32	±	4	[	1.0	]
		2500	24	±	3	[	0.9	]	32	±	5	[	1.0	]
		5000	24	±	3	[	0.9	]	31	±	3	[	1.0	]
TA100	GST	0	116	±	2				129	±	7			
		312.5	113	±	5	[	1.0	]	130	±	5	[	1.0	]
		625	119	±	3	[	1.0	]	132	±	4	[	1.0	]
		1250	117	±	3	[	1.0	]	133	±	6	[	1.0	]
		2500	115	±	5	[	1.0	]	130	±	4	[	1.0	]
		5000	117	±	5	[	1.0	]	131	±	4	[	1.0	]
TA1535	GST	0	10	±	3				11	±	2			
		312.5	12	±	2	[	1.3	]	8	±	1	[	0.7	]
		625	9	±	1	[	1.0	]	10	±	2	[	0.9	]
		1250	10	±	2	[	1.1	]	10	±	3	[	0.9	]
		2500	10	±	1	[	1.1	]	11	±	3	[	1.0	]
		5000	10	±	2	[	1.1	]	11	±	3	[	0.9	]
TA1537	GST	0	9	±	2				10	±	2			
		312.5	11	±	1	[	1.1	]	10	±	2	[	1.0	]
		625	14	±	1	[	1.5	]	12	±	2	[	1.2	]
		1250	11	±	2	[	1.1	]	9	±	1	[	0.9	]
		2500	10	±	2	[	1.1	]	12	±	3	[	1.2	]
		5000	13	±	2	[	1.4	]	11	±	2	[	1.1	]
WP2*uvrA*	GST	0	45	±	3				54	±	3			
		312.5	45	±	3	[	1.0	]	55	±	5	[	1.0	]
		625	43	±	3	[	1.0	]	55	±	3	[	1.0	]
		1250	44	±	4	[	1.0	]	56	±	3	[	1.0	]
		2500	44	±	3	[	1.0	]	57	±	2	[	1.1	]
		5000	46	±	2	[	1.0	]	54	±	4	[	1.0	]
Positive controls														
TA98	AF-2	0.1	480	±	16	[	18.2	]						
TA100	AF-2	0.01	536	±	21	[	4.6	]						
TA1535	NaN_3_	0.5	405	±	46	[	41.9	]						
TA1537	9-AA	40.0	208	±	14	[	22.3	]						
WP2*uvrA*	AF-2	0.01	365	±	24	[	8.1	]						
TA98	2-AA	2.5							287	±	5	[	9.0	]
TA100	2-AA	2.5							1085	±	103	[	8.4	]
TA1535	2-AA	2.0							344	±	4	[	30.4	]
TA1537	2-AA	2.0							224	±	38	[	23.2	]
WP2*uvrA*	2-AA	10.0							400	±	22	[	7.4	]

Values are presented as the mean ± S.D.

1 No. of colonies of treated plate/No. of colonies of negative control plate.

AF-2, 2-(2-furyl)-3-(5-nitro-2-furyl) acrylamide; NaN_3_, sodium azide; 9-AA, 9-aminoacridine hydrochloride monohydrate; and 2-AA, 2-aminoanthracene.

**Table 2.** Chromosome analysis of GST in Chinese hamster lung fibroblast cells with and without metabolic activation (S9 mixture)

Treatment time	S9 mixture	Concentration (μg/mL)	GST	
			% numerical aberration	% structural aberration (Exclusive to gap)
6 h	-	0	0.0	0.0
		12.5	0.0	0.0
		25	0.0	0.3
		50	0.0	0.0
		70	-	-
		90	-	-
		Positive control^a^	0.0	13.7 ^*^
6 h	+	0	0.0	0.0
		20	0.0	0.0
		40	0.0	0.0
		80	0.0	0.3
		100	-	-
		120	-	-
		Positive control^b^	0.0	16.3^*^
24 h	-	0	0.0	0.0
		7.5	0.0	0.3
		15	0.0	0.0
		30	0.0	0.0
		50	-	-
		70	-	-
		Positive control^a^	0.0	17.0 ^*^

a Mitomycin C;

b Cyclophosphamide monohydrate

* *P* < 0.01 compared with the negative control group.

**Table 3.** Frequencies of MNPCE per 2,000 PCE in the bone marrow of ICR mice exposed to GST

Sex	Chemical treated	Dose(mg/kg)	No. of animal	MNPCE/2000 PCE			PCE/(PCE+NCE)		
Male	Vehicle	0	5	0.08	±	0.02	50.03	±	1.43
	GST	500	5	0.06	±	0.01	50.60	±	1.98
	GST	1000	5	0.05	±	0.04	48.60	±	0.68
	GST	2000	5	0.05	±	0.04	49.18	±	0.26
	CPA	70	5	6.69	±	0.37 ^*^	45.83	±	0.89 ^*^

Values are presented as the mean ± S.D. (%).

* *P* < 0.01 compared with the negative control group.

CPA, cyclophosphamide monohydrate; MNPCE, micronucleated polychromatic erythrocyte; NCE, normochromatic erythrocyte; and PCE, polychromatic erythrocyte.

**Table 4.** Body weights of ICR mice exposed to GST

Sex	Chemical treated	Dose (mg/kg)	Body weights at the time of								
			Administration						Sacrifice		
			1st			2nd					
Male	Vehicle	0	34.60	±	1.28	34.00	±	1.47	33.81	±	1.15
	GST	500	34.94	±	1.17	34.81	±	1.34	34.91	±	1.18
	GST	1000	34.82	±	1.00	34.53	±	1.16	34.23	±	1.02
	GST	2000	34.46	±	0.91	34.57	±	0.76	34.16	±	0.80
	CPA	70	34.63	±	1.16	34.20	±	1.02	33.43	±	0.96

Values are presented as the mean ± S.D. (g). CPA, Cyclophosphamide monohydrate
